# British Hospitals Association

**Published:** 1921-02-26

**Authors:** 


### British Hospitals Association.

### MEETING AT NOTTINGHAM OF EAST MIDLANDS BRANCH.

A largely attended meeting of this Association was held at the Nottingham General Hospital on February 16, Mr. Frederick Acton presiding. Representatives of the voluntary hospitals of Nottinghamshire, Derbyshire, Leicestershire, Northamptonshire, Lincolnshire, and Rutlandshire attended.

Mr. Frederick Acton, C.B.E., Chairman of the Nottingham General Hospital, was elected Chairman, and Mr. E. C. Barnes, Chairman of the Chesterfield Royal Infirmary, Vice-Chairman, for the ensuing year. Mr. T. Fielding Johnson, Chairman of the Leicester Royal Infirmary, was elected as representative of the branch on the Council of the British Hospitals Association. Mr. Walter Banks, Superintendent and Secretary of the Derbyshire Royal Infirmary, was elected Honorary Secretary.

A long discussion took place on the College of Nursing scale of salaries for the nursing staffs of the voluntary hospitals. Mr. Acton warmly advocated the adoption of a national scheme of superannuation for nurses. It was resolved that further consideration of the scale of salaries be postponed until a future meeting.

On the motion of Mr. Harry Johnson (Leicester) it was resolved that the Council of the British Hospitals Association be asked to obtain from the Inland Revenue authorities particulars as to what concessions are at present given to subscribers to voluntary hospitals in respect of contributions, with a view to obtaining further concessions in place of those granted under Excess Profits Duty, while on the motion of Mr. Barnes, of Chesterfield, it was resolved that the Council of the Association be requested to urge the Government to make a substantial concession to voluntary hospitals in respect of telephone charges.

It was decided that it is advisable that this branch of the British Hospitals Association be represented on the Government inquiry on the position of the voluntary hospitals. The following hospitals were chosen to be represented on the inquiry, together with such others as the Committee of Inquiry may select: Buxton Devonshire Hospital, Chesterfield Royal Infirmary, Derbyshire Royal Infirmary, Grantham Hospital, Leicester Royal Infirmary, Lincoln County Hospital, Newark District Hospital, Northampton General Hospital, Nottingham General Hospital, together with Mr. Walter Banks, as representing the smaller hospitals of the branch.

On the suggestion of Mr. Harry Johnson it was decided that application for exemption of the voluntary hospitals from regulations of the Dangerous Drugs Act be made to the Home Secretary.

An Executive Committee was appointed, consisting of representatives of the Chesterfield Royal Infirmary, Derbyshire Royal Infirmary, Leicester Royal Infirmary, Lincoln County Hospital, and the Nottingham General Hospital.

